# Association between Reproductive Factors and Age-Related Macular Degeneration in Postmenopausal Women: The Korea National Health and Nutrition Examination Survey 2010-2012

**DOI:** 10.1371/journal.pone.0102816

**Published:** 2014-07-15

**Authors:** Bum-Joo Cho, Jang Won Heo, Jae Pil Shin, Jeeyun Ahn, Tae Wan Kim, Hum Chung

**Affiliations:** 1 Department of Ophthalmology, Seoul National University College of Medicine, Seoul, Korea; 2 Department of Ophthalmology, Seoul National University Hospital, Seoul, Korea; 3 Department of Ophthalmology, Kyungpook National University School of Medicine, Daegu, Korea; 4 Department of Ophthalmology, Seoul Metropolitan Government Seoul National University Boramae Medical Center, Seoul, Korea; Massachusetts Eye & Ear Infirmary, Harvard Medical School, United States of America

## Abstract

**Purpose:**

To examine the association between female reproductive factors and age-related macular degeneration (AMD) in postmenopausal women.

**Design:**

Nationwide population-based cross-sectional study.

**Methods:**

A nationally representative dataset acquired from the 2010–2012 Korea National Health and Nutrition Examination Survey was analyzed. The dataset involved information for 4,377 postmenopausal women aged ≥50 years with a fundus photograph evaluable for AMD in either eye. All participants were interviewed using standardized questionnaires to determine reproductive factors including menstruation, pregnancy, parity, lactation, and hormonal use. The association between reproductive factors and each type of AMD was investigated.

**Results:**

The mean age of the study participants was 63.1±0.2 years. Mean ages at menarche and menopause were 16.1±0.0 and 49.2±0.1 years, respectively. The overall prevalence rates of early and late AMD were 11.2% (95% confidence interval [CI], 10.1–12.5) and 0.8% (95% CI, 0.5–1.2), respectively. When adjusted for age, neither smoking nor alcohol use was associated with the presence of any AMD or late AMD. Multivariate logistic regression analysis revealed age (OR, 1.12 per 1 year), duration of lactation (OR, 0.91 per 6 months), and duration of use of oral contraceptive pills (OCP) (OR, 1.10 per 6 months) as associated factors for late AMD. The other variables did not yield a significant correlation with the risk of any AMD or late AMD.

**Conclusion:**

After controlling for confounders, a longer duration of lactation appeared to protect against the development of late AMD. A longer duration of OCP use was associated with a higher risk of late AMD.

## Introduction

The leading cause of blindness and visual impairment in elderly individuals of developed countries is age-related macular degeneration (AMD) [1], [2]. With the expanding human lifespan, AMD has garnered increasing interest among researchers. Several risk factors have been identified for this condition, including cigarette smoking, hyperopia, and genetic variation in complement factor H [3]–[5].

Among those risk factors, female sex has been associated with a higher prevalence of AMD in many population-based studies [6]. Recent researches have suggested gender differences in the pathophysiology of AMD arising from dissimilar hormonal status [5], [6]. However, there have not been sufficient data on the association between female own risk factors and AMD. Only a few epidemiological studies were performed, and the results have been inconclusive to date [7]–[10]. Identification of female risk factors for AMD would help to understand the pathogenesis of the disease and screen the patients at risk. Because most female patients at risk for AMD are post-menopausal, the associated hormonal changes must be examined closely as well.

Therefore, in this study, we investigated the association between female reproductive factors and each type of AMD in postmenopausal women. Various factors related to menstruation, pregnancy, delivery, lactation, and the use of hormonal drugs were explored. To acquire representative data for the general population, we analyzed the nationwide data obtained as part of the Korea National Health and Nutrition Examination Survey (KNHANES) on behalf of the Korean Ophthalmological Society (KOS) [11]. The ethnic homogeneity of Korea might facilitate to reveal the risk factors of AMD by minimizing the bias resulting from interracial differences [12]. To the authors’ knowledge, this is the first study to analyze this association in an Asian population.

## Materials and Methods

### Study population

The data analyzed in this study were obtained from the fifth cycle of the KNHANES which was performed from 2010 through 2012. The KNHANES is an ongoing nationally representative cross-sectional survey to examine the health, physical, and nutritional status of the general Korean population [11], [12]. The survey was first administered in 1998 and has been conducted annually since 2007 by the Korea Center for Disease Control and Prevention (KCDC). Ophthalmologic examinations were included since the second half of 2008. Details relating to the KNHANES design and methods have been presented elsewhere [11], [12]. To summarize briefly, the study methodology involves stratified multistage cluster-sampling to prevent subject omission or overlap. The rolling-sampling method makes each annual survey results representative for the entire Korean population and mergeable with the past results. During the period from 2010–2012, the KNHANES annually included 3,800 households from 192 enumeration districts. From each household, all family members aged ≥1 year were included as eligible subjects [11]. The eligible subjects were asked to take part in health interviews and physical examinations including comprehensive ophthalmologic assessments in mobile centers by trained teams. Non-mydriatic 45° color fundus photographs were obtained for each subject aged ≥19 years in a dark room using a digital fundus camera (TRC-NW6S; Topcon, Tokyo, Japan). When the non-mydriatic photograph was of insufficient quality for grading due to media opacity or a small pupil, mydriatic fundus photographs were obtained at the point of maximal pupillary dilation, with the patients’ consent.

Among the participants, only postmenopausal women were included in this study. As suggested previously, premenopausal women, women aged <50 years, women who experienced menopause before the age of 30, or those who did not report the age of menopause were excluded [10], [13]. Subjects without any fundus photograph evaluable for the presence of AMD were also excluded from this study. Ultimately, only postmenopausal women who were aged ≥50 years and who had ≥1 assessable fundus photograph were included. The study described here adhered to the tenets of the Declaration of Helsinki, and written informed consent was obtained from all participants. The survey protocol was approved by the Institutional Review Board of the KCDC (IRB No: 2010-02CON-21-C, 2011-02CON-06-C, 2012-01EXP-01-2C).

### Assessment of Reproductive factors

Female reproductive factors, demographic variables, and health behavioral factors were assessed on the basis of self-reported answers to a standardized questionnaire. The reproductive factors evaluated included the following: age at menarche, age at menopause, type of menopause, number of pregnancies, number of spontaneous and/or artificial abortions, parity (the number of children given birth to), lactation, use of oral contraceptive pills (OCP), and use of postmenopausal female hormone replacement therapy (HRT).

The type of menopause was dichotomized as natural vs. artificial (e.g., hysterectomy or oophorectomy). The duration of lactation, OCP use, or HRT use was recorded as the total number of experienced months. The drug components of HRT and/or OCP were not specified. Length of the reproductive period was calculated as follows: age at menopause – age at menarche. Duration of the postmenopausal period was designated as follows: age – age at menopause. Duration of lactation per child was calculated from the division of duration of lactation by the number of parity.

### Assessment of AMD

The presence of each AMD type was determined on the fundus photographs [11]. Each fundus photograph was preliminarily evaluated for the presence of AMD on site by dispatched ophthalmologists who were trained for grading by the National Epidemiologic Survey Committee of the KOS and used the International Age-related Maculopathy Epidemiological Study Group grading system [14]. Detailed grading was later performed by nine retina specialists with experience in grading AMD, who were masked to the patients’ characteristics. Any discrepancy between the preliminary and detailed grading was resolved by an independent ophthalmologist (J.P.S.). Drusen were classified on the basis of size, appearance, and edge sharpness [14]. Retinal pigmentary abnormalities were graded as hypo- or hyperpigmentation [14]. Patients were defined as having early AMD if they met any one of the following criteria: (1) the presence of soft indistinct drusen or reticular drusen; (2) the presence of hard or soft distinct drusen with pigmentary abnormalities in the absence of late AMD [14]. Late AMD was defined as either the presence of neovascularization or geographic atrophy [14]. AMD was classified as neovascular if associated with detachment of the retinal pigment epithelium (RPE), serous detachment of the neurosensory retina, subretinal or sub-RPE hemorrhages, or subretinal fibrous scars [14]. Geographic atrophy was defined as a circular area with a sharp edge ≥175 µm in diameter showing hypopigmented RPE and apparent choroidal vessels, in the absence of signs for neovascular AMD [14]. When the severity of AMD differed between eyes, the subject was assigned the more advanced grade, and when only one eye could be assessed, the subject was assigned the grade of that eye. The presence of any AMD was defined as having either early AMD or late AMD. The quality of the grading was verified by the KOS. Grading agreement between the preliminary graders and the standard reading specialists ranged from 94.1–96.2%.

### Statistical Analysis

Statistical estimations were performed using the sampling weights adjusted for response rate, extraction rate, and the distribution of the general Korean population. Continuous variables were expressed as mean ± standard error or mean with 95% confidence intervals (CIs). Categorization was performed for some of the continuous variables. In order to assess the association with AMD, the odds ratios (ORs) of continuous and categorical variables were calculated.

Prior to main analyses, the confounders for the risk of AMD were investigated among demographic and health behavioral variables by age-adjusted univariate logistic regression analyses. Next, univariate logistic regression analyses were performed to screen the potential reproductive risk factors for any AMD or late AMD after controlling for confounders. Risk factors with P<0.1 were selected, and multicollinearity among them was examined by calculating the variance inflation factors (VIFs). Those with a VIF ≥5 were excluded from subsequent analyses. Finally, multivariate logistic regression analyses were performed using a stepwise selection method. Final models for the presence of AMD were constructed using the set of risk factors with P<0.05 in the multivariate analysis. All statistical analyses were performed using SPSS 20.0 for Windows (SPSS Inc., Chicago, IL).

## Results

**Figure 1:**
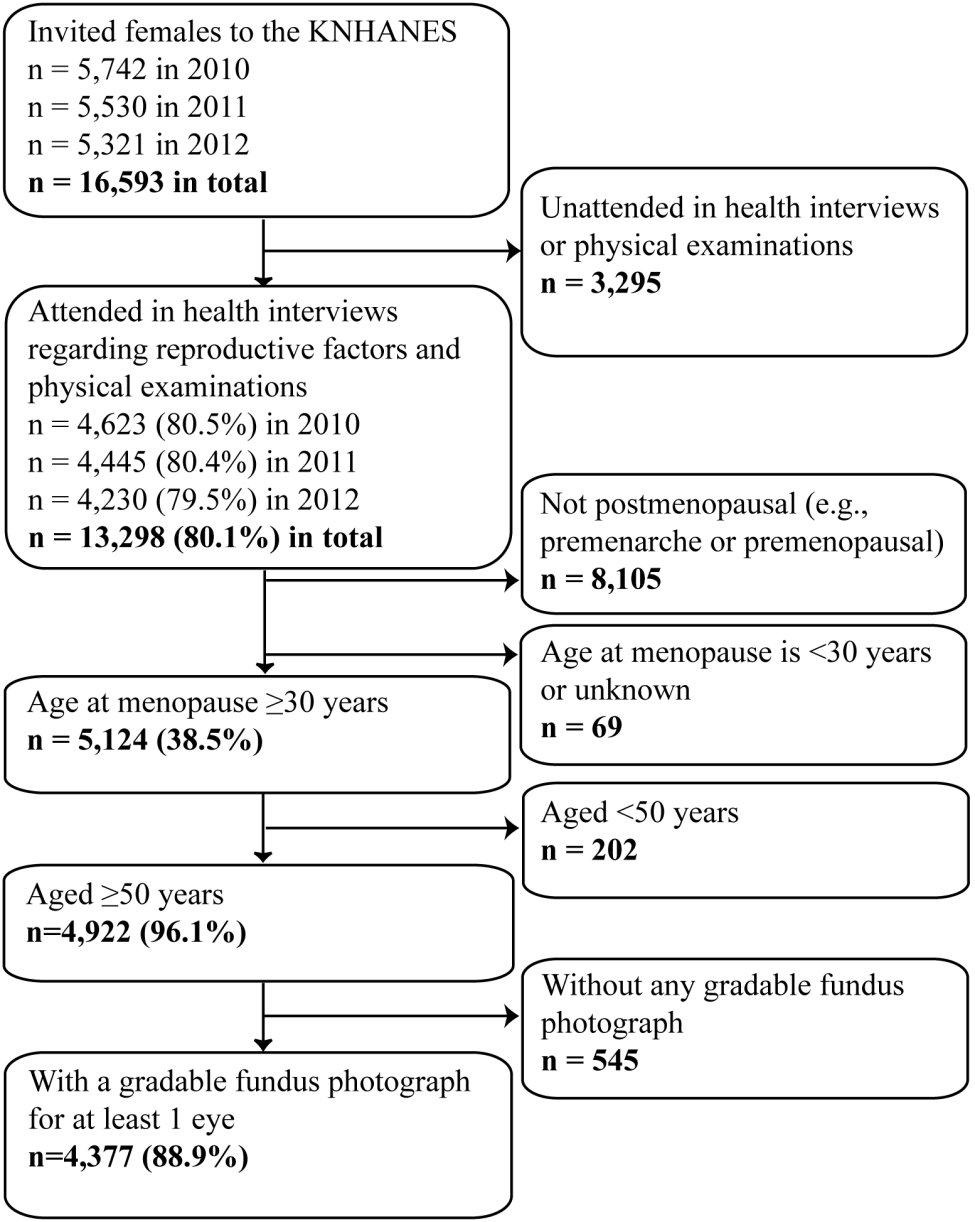
Participation flow-chart for the Korea National Health and Nutrition Examination Survey (KNHANES) during the period from 2010–2012.

During the period from 2010–2012, among 16,593 eligible women, 13,298 women were interviewed and underwent physical examinations (response rate, 80.1%) (Fig. 1). Participation rates during the period from 2010–2012 ranged from 79.5–80.5%. Of these, 4,922 subjects were postmenopausal and aged ≥50 years old. Among these, 4,377 (88.9%) subjects with a fundus photograph for either eye that could be used to assess the presence of AMD were ultimately included in this study.

### Demographics of Study Participants

The mean age of the all study participants was 63.1±0.2 years (range, 50–97 years). Mean ages at menarche and menopause were 16.1±0.0 years (range, 8–28 years) and 49.2±0.1 years (range, 30–72 years), respectively. The age-stratified reproductive characteristics of the study group are presented in Table 1. In younger generations, reproductive years were longer and the numbers of pregnancy and parity were smaller compared to those in older generations (P<0.001 for all). Duration of lactation was shorter in the younger generations than in the older generations (P<0.001), while both the periods of OCP use and HRT use tended to be longer in the younger generations than in the older generations (P<0.001).

**Table 1:** Age-based stratification of postmenopausal women aged ≥50 years in the KNHANES 2010–2012.

	50–59^a^(n = 1586)	60–69^a^(n = 1516)	70–79^a^(n = 1080)	≥80^a^(n = 195)	P Value
Age, y	54.6±0.1	64.3±0.1	74.1±0.1	82.6±0.2	<0.001^b^
Prevalence of early AMD, %	4.8±0.7	11.8±1.0	20.7±1.7	22.2±4.0	<0.001^c^
Prevalence of late AMD, %	0.3±0.2	0.7±0.3	1.1±0.3	3.4±1.6	0.002^c^
Age at menarche, y	15.7±0.1	16.3±0.1	16.7±0.1	16.6±0.2	<0.001^b^
Age at menopause, y	49.4±0.1	49.9±0.2	48.0±0.2	47.7±0.5	<0.001^b^
Reproductive years, y	33.7±0.1	33.6±0.2	31.3±0.2	31.2±0.5	<0.001^b^
Postmenopausal period, y	5.2±0.1	14.4±0.2	26.1±0.2	34.9±0.5	<0.001^b^
Number of pregnancy	3.9±0.1	5.0±0.1	6.2±0.1	6.2±0.2	<0.001^b^
Number of spontaneous abortion	0.3±0.0	0.3±0.0	0.3±0.0	0.3±0.1	0.988^b^
Number of artificial abortion	1.3±0.0	1.5±0.1	1.4±0.1	0.6±0.1	<0.001^b^
Number of parity	2.3±0.0	3.2±0.0	4.5±0.1	5.2±0.2	<0.001^b^
Duration of lactation, m	25.2±0.8	50.0±1.2	90.8±2.1	107.9±5.4	<0.001^b^
Duration of OCP use, m	4.1±0.6	7.6±0.7	6.0±0.7	1.0±0.5	<0.001^b^
Duration of HRT use, m	5.3±0.5	7.4±0.8	1.8±0.5	0.9±0.6	<0.001^b^

AMD = age-related macular degeneration; OCP = oral contraceptive pills; HRT = hormone replacement therapy.

a Age in years.

b General linear model for complex samples.

c Pearson’s chi-square test for complex samples.

The overall prevalence rates of early, late, and any AMD among the study participants were 11.2% (95% CI, 10.1–12.5), 0.8% (95% CI, 0.5–1.2), and 12.0% (95% CI, 10.8–13.2), respectively. The overall prevalence of neovascular AMD and geographic atrophy was 0.6% (95% CI, 0.4–1.0) and 0.1% (95% CI, 0.0–0.5).

Data pertaining to demographic and reproductive characteristics are presented according to the type of AMD in Table 2. Both early AMD subjects and late AMD subjects were significantly older than those without any AMD (P<0.001 and P = 0.006, respectively). Postmenopausal period was also significantly longer in early AMD subjects and in late AMD subjects than in those without any AMD (P<0.001 and P = 0.010, respectively).

**Table 2:** Demographics of postmenopausal women aged ≥50 years by the type of age-related macular degeneration (AMD) in the KNHANES 2010–2012.

	No AMD(n = 3845)	Early AMD		Late AMD	
		(n = 500)	P value^a^	(n = 32)	P value^a^
Age, y	62.4±0.2	68.9±0.5	<0.001	69.6±2.7	0.006
Age at menarche, y	16.1±0.0	16.4±0.1	0.005	16.9±0.4	0.084
Age at menopause, y	49.2±0.1	48.9±0.3	0.242	48.8±0.9	0.661
Reproductive years, y	33.1±0.1	32.5±0.3	0.044	32.0±0.8	0.141
Postmenopausal period, y	13.2±0.2	20.0±0.6	<0.001	20.8±3.0	0.010
Number of pregnancy	4.7±0.0	5.5±0.1	<0.001	4.4±0.4	0.369
Number of spontaneous abortion	0.3±0.0	0.3±0.0	0.657	0.2±0.1	0.220
Number of artificial abortion	1.3±0.0	1.3±0.1	0.708	1.0±0.4	0.485
Number of parity	3.1±0.0	3.8±0.1	<0.001	3.1±0.2	0.948
Duration of lactation, m	48.4±1.1	69.7±3.1	<0.001	44.5±6.7	0.565
Duration of OCP use, m	5.2±0.4	5.9±0.9	0.495	27.7±21.5	0.296
Duration of HRT use, m	5.0±0.4	4.5±0.9	0.570	4.1±2.6	0.724

OCP = oral contraceptive pills; HRT = hormone replacement therapy.

a Comparison with no AMD group, not adjusted for any covariate.

Univariate binary logistic regression analysis showed that age was highly associated with the presence of any AMD and that of late AMD (OR 1.08; 95% CI, 1.07–1.09 and OR 1.07; 95% CI, 1.02–1.14, respectively). When adjusted for age, the presence of any AMD was not associated with a history of ever smoking, current smoking, or current alcohol use (P = 0.826, P = 0.139, and P = 0.701, respectively). The same trend was observed for late AMD (P = 0.439, P = 0.795, and P = 0.798, respectively).

### Association between Reproductive Factors and AMD

The association of reproductive factors with any AMD and late AMD is summarized in Table 3. Age-adjusted univariate logistic regression analysis did not reveal any associated factor (P<0.1) for the presence of any AMD. On the other hand, age-adjusted univariate logistic regression analysis showed that the following factors were correlated with the presence of late AMD (P<0.1): number of pregnancy, number of parity, duration of lactation per child, duration of lactation, and duration of OCP use. Among these variables, the VIFs of number of parity, duration of lactation per child, and duration of lactation were ≥5 (5.618, 5.025, and 10.000, respectively). Duration of lactation per child was excluded from next analyses, because it was obtained from the calculation with number of parity and duration of lactation, and the authors reached a consensus that it would be a less meaningful variable than total duration of lactation in the development of AMD. After the exclusion, the corresponding VIFs of number of pregnancy, number of parity, duration of lactation, and duration of OCP use were all <5 (1.852, 3.300, 2.538, and 1.009, respectively).

**Table 3:** Age-adjusted odds ratios (ORs) of age-related macular degeneration (AMD) for reproductive risk factors in postmenopausal women in the KNHANES 2010–2012.

Reproductive risk factors	Increment	Any AMD			Late AMD		
		OR	95% CI	P Value	OR	95% CI	P Value
Age at menarche	1 year	1.01	0.96–1.06	0.705	1.13	0.90–1.41	0.289
Age at menopause	1 year	1.00	0.98–1.03	0.746	1.00	0.94–1.07	0.911
Reproductive years	1 year	1.00	0.98–1.02	0.878	0.99	0.94–1.04	0.634
Postmenopausal period	1 year	1.00	0.97–1.02	0.744	1.00	0.94–1.06	0.908
Number of pregnancy	1	1.00	0.95–1.04	0.911	0.81	0.67–0.97	0.025
Number of spontaneous abortion	1	0.97	0.82–1.14	0.677	0.79	0.47–1.32	0.364
Number of artificial abortion	1	1.00	0.94–1.06	0.963	0.92	0.64–1.32	0.644
Number of parity	1	1.00	0.92–1.08	0.999	0.74	0.62–0.88	0.001
Duration of lactation per child	1 month	1.00	0.98–1.01	0.415	0.96	0.91–1.00	0.053
Duration of lactation^a^	6 months	1.00	0.98–1.01	0.616	0.91	0.87–0.95	<0.001
Duration of OCP use^a^	6 months	1.03	0.99–1.07	0.149	1.09	1.01–1.18	0.025
Duration of HRT use^a^	6 months	1.01	0.99–1.04	0.436	1.00	0.93–1.09	0.881
Artificial menopause	Yes	0.92	0.62–1.37	0.667	0.87	0.26–2.91	0.818
Bilateral oophorectomy	Yes	1.08	0.61–1.90	0.798	1.66	0.45–6.16	0.449

CI = confidence interval; OCP = oral contraceptive pills; HRT = hormone replacement therapy.

a Total years of experience.

In a multivariate logistic regression analysis using these variables, number of pregnancy and number of parity yielded insignificant correlations. Ultimately, the final regression model for late AMD included age (OR, 1.12 per year; 95% CI, 1.06–1.18), duration of lactation (OR per 6 months, 0.91; 95% CI 0.86–0.95), and duration of OCP use (OR, 1.10 per 6 months; 95% CI, 1.02–1.18) as associated factors. Among these, duration of lactation was shown to protect against late AMD, with a 9% risk reduction per 6-month breast-feeding. The other factors were positively correlated with the risk of late AMD (Table 4).

**Table 4:** Multivariate-adjusted odds ratios (ORs) of late age-related macular degeneration (AMD) for reproductive risk factors in postmenopausal women in the KNHANES 2010–2012.

	Risk factors	Increment	OR	95% CI	P Value
Model 1	Age	1 year	1.12	1.06–1.17	<0.001
	Number of pregnancy	1	0.82	0.53–1.28	0.386
	Number of parity	1	1.11	0.61–2.02	0.725
	Duration of lactation^a^	6 months	0.92	0.85–1.00	0.043
	Duration of OCP use^a^	6 months	1.10	1.02–1.19	0.013
Model 2^b^	Age	1 year	1.12	1.06–1.18	<0.001
	Duration of lactation^a^	6 months	0.91	0.86–0.95	<0.001
	Duration of OCP use^a^	6 months	1.10	1.02–1.18	0.019

CI = confidence interval; OCP = oral contraceptive pills.

a Total years of experience.

b Final multivariate model consisted of risk factors of p value<0.05.

## Discussion

The present study examined the association of various reproductive factors with the risk of AMD, in a representative population of postmenopausal Korean women. The investigated factors included menstruation, pregnancy, abortion, parity, lactation, as well as the use of OCP and HRT. After controlling for confounders, the duration of lactation was inversely associated with the risk of late AMD. In contrast, the use of OCP increased the risk of late AMD.

An interesting finding in this study is that breast-feeding has a protective effect against late AMD. More specifically, a 6-month increment in the total duration of lactation was associated with a 9% decrease in the risk of late AMD. This finding was first suggested by Erke et al in a recent research [7]. The authors stated that an increase in the total duration of lactation was significantly associated with a reduced risk of late AMD (OR per 3 months, 0.84) and a 1-month increase in the duration of lactation per child decreased the risk of late AMD by 20% [7]. The current study consisted with this result and identified the protective effect in a period-dependent manner. The amount of protective effect was less than that in the previous study [7]. Thus far, no other prior study has explored the association between lactation and AMD in the literature. Notably, the duration of lactation appeared to be significantly higher in the early AMD group in this study compared to that in the control group. However, this difference was eliminated in the regression analysis after age-adjustment, indicating that the increase in the duration of lactation in the early AMD group may have arisen from the increased age of the group.

The mechanism underlying the association between lactation and AMD is not well understood, but it might be approached in consideration of the protective effect of breast-feeding against several cardiovascular diseases [7], [15]. In Women’s Health Initiative study that included 139,681 postmenopausal women, those who had breastfed for >12 months were less likely to have hypertension (OR, 0.88), diabetes mellitus (OR, 0.80), hyperlipidemia (OR, 0.81), and cardiovascular disease (OR, 0.91) than those who had never breastfed [15]. In a Norwegian prospective population-based cohort study, lactation for ≥24 months decreased the cardiovascular mortality significantly among parous women aged <65 years (hazard ratio, 0.36) [16]. Additionally, lactation is known to improve the subclinical vascular indices of cardiovascular disease [17], to promote lipid metabolism [18], and to reduce serum concentrations of C-reactive protein [19]. Moreover, these effects were found to be long-lasting in a large cohort study [20]. Late AMD has been associated with cardiovascular diseases in several studies [21], [22], thus the protective effect of lactation against cardiovascular diseases might also help to reduce the risk of late AMD. This might be mediated by the effects on the microvasculature of choroid or retina. On the other hand, lactation has an inhibitory effect on the female hormonal axis, including estrogen exposure [15]. Considering that estrogen exposure has shown a protective effect against development of AMD in some previous studies [23], the protective effect of lactation against late AMD described above may override the anti-protective effect mediated by the inhibition of the hormonal axis.

Another novel finding presented here is the positive correlation between the use of OCP and the risk of late AMD. The present study showed a 10% increase in the risk of late AMD per 6-month increase of OCP use. Thus far, there have been only a few studies on the association between AMD and OCP use [5], [7], [10], [24]. One study reported a reduced risk of neovascular AMD (OR, 0.55) in women who had used OCP at least once [5], and another found a decreased the risk of early AMD (OR, 0.5) in those who had used OCP ever [24]. Other researchers have reported that the use of OCP was not associated with the risk of early or late AMD in postmenopausal women [7], [10]. On the other hand, since the introduction of OCP, a variety of vascular complications have been reported to be associated with it [25]. The risky diseases associated with OCP include deep-vein thrombosis, pulmonary embolism, stroke, and myocardial infarction (MI) [25]. A recent meta-analysis revealed a three-fold increase in the risk of venous thromboembolism, a two-fold increase in the risk of ischemic stroke, and an indeterminate effect on the risk of MI [25]. The positive association between OCP use and late AMD in this study is in line with the increased rate of cardiovascular disease observed for OCP users.

The pathophysiology of the association of OCP with cardiovascular diseases or late AMD is not clearly elucidated yet. OCP are typically either a combination of progestin and lower doses of estrogen or progestin only [26]. OCP increase the levels of prothrombin fragments, fibrinogen, plasmin–antiplasmin complex, and protein C activity, and decrease antithrombin activity and the level of tissue-plasminogen activator [27], [28]. These hemostatic effects are known to be modulated by the progestin’s potency as an androgen [27]. Progestin is also suggested to increase the level of aminopeptidase P and the breakdown of bradykinin, and thereby to increase blood pressure [29]. The hemodynamic and hemostatic effects of OCP might contribute to the risk of cardiovascular disease, and thus affect the development of AMD. However, further studies with larger sample sizes will be necessary in the future to validate the findings of this study.

Estrogen is considered one of the most important reproductive hormones in women. Endogenous exposure to estrogen starts with the release of estrogen from the ovary, and is related to the age at menarche, age at menopause, and the number of pregnancies [23]. The Aravind Comprehensive Eye Survey showed age at menarche ≥14 years, which means the late start of estrogen release, is a risk factor for overall AMD (OR, 2.3) [30], and the Rotterdam Study showed an increased risk of AMD in those who experienced early menopause following oophorectomy (OR, 3.8) [3]. The Blue Mountain Eye Study reported that a long reproductive period was associated with a decreased prevalence of early AMD [31]. These findings provide further support for the notion that a shorter duration of estrogen exposure may increase the risk of AMD. However, no such association was found in this study.

The association between AMD and exogenous exposure to estrogen in the form of HRT has been inconsistent in previous studies [5], [9], [13], [23], [32]. A 34% increase in the risk of early AMD was observed among current HRT users in one report as compared to that in individuals who had never used HRT [10]. To the contrary, HRT was associated with a lower risk of neovascular AMD (RR, 0.52) among female nurses aged 30–55 years [10], and reduced the risk of exudative AMD (OR, 0.6) in the Eye Disease Case–Control Study [33]. A postulated explanation for this association is that estrogen deficiency results in a down-regulation of matrix metalloproteinase–2 activity and an up-regulation of YKL-40 protein which may accelerate choroidal neovascularization [24], [34]. However, most studies to date have reported no association between estrogen treatment and early or late AMD [3], [7], [8], [24], [30], [32]. In the current study, we were also unable to find an association between HRT and any AMD or late AMD. This result might stem from our limited sample size.

The present study has several limitations. As it was a cross-sectional survey, the association between risk factors and late AMD does not guarantee causality. The result means only a cross-sectional distribution of AMD in a certain population, and a survivorship bias may intervene. Therefore, a longitudinal cohort study is required to validate the findings presented here and disclose the causality. It will also help to examine the incidence of AMD in the population at risk. In addition, as the information for several reproductive factors in this survey was based on self-reported answers from the participants, and not on their medical records, there could be an intervening recall bias. To minimize biases, the health interviews in this survey were performed using a standardized questionnaire by trained teams. However, because most participants were old, there may be some recall bias remaining in this study. Moreover, although we tried to control for several potential confounders, the possibility of a confounding effect between the risk factors studied and AMD still remains. The small number of late AMD patients represents another limitation that may have reduced the statistical power of our conclusions. Regarding OCP use, more specific data such as the drug components were not investigated in the current survey. Future studies involving the drug components of OCP are necessary in order to reveal the association of OCP use with AMD more clearly. Lastly, subjects who could not have an evaluable fundus photograph taken due to mature cataract or other media opacity were excluded. Because these patients tended to be older [12] and thus were more likely to have had AMD, the prevalence of AMD may have been underestimated. The strength of this study derives largely from our use of data from a large, nationally representative sample population.

In conclusion, the current study suggests a longer period of lactation might protect against late AMD. We also presented our novel finding that a longer use of OCP increases the risk of late AMD. These findings could elucidate the development of AMD and help the screening of patients at risk as well as the prevention of AMD among postmenopausal women in public health.
